# Assessment of knowledge and use of HIV primary and secondary prevention strategies in Portugal: a scoping review

**DOI:** 10.1186/s12889-026-27542-7

**Published:** 2026-05-07

**Authors:** João Brázia, Boxuan Wang, Paula Meireles, Eugenio Valdano, Andreia Sofia Teixeira

**Affiliations:** 1 BRAN Lab, Network Science Institute, Northeastern University London, Devon House, 58 St Katharine’s Way, London, E1W 1LP UK; 2 Faculdade de Ciências, LASIGE, Universidade de Lisboa, Lisboa, Portugal; 3 Sorbonne Université, INSERM, Institut Pierre Louis d’Epidémiologie et de Santé Publique, Paris, F75012 France; 4 EPIUnit ITR, Instituto de Saúde Pública da Universidade do Porto, Universidade do Porto, Rua das Taipas, n° 135, Porto, 4050-600 Portugal

**Keywords:** HIV, Scoping review, Knowledge, Use, Assessment, Key population

## Abstract

**Background:**

Portugal has been committed to achieve UNAIDS target goals by the end of 2030 by improving accessibility of prevention programs to the general population and key populations. However, Portugal remains as one of the countries in the European Union with the highest annual number of reported HIV cases and the highest proportion of AIDS diagnosis. The main aim of this scoping review is to understand how knowledge and usage of primary and secondary prevention strategies are being assessed in Portugal across key populations, such as sex workers, men who have sex with men, people who inject drugs, migrants and transgender.

**Methods:**

We performed searches on PubMed, Web of Science Core Collection and Scopus for peer-reviewed journal articles published in English or in Portuguese, between 2008 and 2025, that reported knowledge and/or use assessment of at least one prevention outcome (condom, HIV testing, PrEP, PEP) in at least one of the key population groups under study.

**Results:**

From the 655 studies identified in our initial search, 54 met the eligibility criteria for inclusion. Most studies focused on condoms and testing. While we did not find studies focused on condom knowledge assessment, condom usage was characterized using a broad set of indicators defined according to the type of partnership, gender, type of sexual intercourse, sexual role preference, time window, and frequency across key populations. Knowledge about HIV testing was defined across studies as knowing where to get tested and/or that HIV testing is confidential and free, while usage was defined for multiple frequencies and recall periods. PrEP and PEP knowledge and usage were often assessed coupled with condom usage in the last 6 months.

**Discussion:**

The assessment of primary and secondary prevention strategies was highly heterogeneous. Multiple instrument assessment methods were defined for different recall periods, which makes it challenging to compare assessment methods across studies, both within and across key populations, and to develop mathematical modelling studies that can inform public health policies.

**Conclusion:**

This review highlights the need to promote the homogeneity and harmonization of studies within and between key populations, which is crucial to develop successful HIV prevention programs in Portugal.

**Supplementary Information:**

The online version contains supplementary material available at 10.1186/s12889-026-27542-7.

## Background

Since the signature of the Dublin declaration in 2004 [1], Portugal has made, together with other countries in western Europe, substantial progress in the fight against the HIV/AIDS epidemic. According to the latest European Centre for Disease Prevention and Control (ECDC) report, Portugal has already reached the first of the three Joint United Nations Programme on HIV/AIDS (UNAIDS) 95-95-95 targets adopted in 2021 to end the AIDS (Acquired Immunodeficiency Syndrome) epidemic by 2030 [2]: to ensure that at least 95% of people living with HIV know their status, 95% of those who know their status are on antiretroviral therapy (ART), and 95% of those on ART achieve viral suppression.

Improvements in primary prevention have allowed Portugal to reduce HIV acquisitions by 37% from 2014 to 2023. Notwithstanding, Portugal still has one of the highest rates of HIV diagnosis in the European Union (EU) (8.5 infection cases per 105 inhabitants for Portugal vs. 5.1 in the EU overall, in 2022), and HIV diagnosis at the AIDS stage is also three times more likely (1.6 infection cases per 105 inhabitants for Portugal vs. 0.5 in the EU overall, in 2022) [3, 4]. These figures show the need for Portugal to improve both primary and secondary prevention of HIV. Primary prevention aims to reduce the risk of HIV acquisition [5] and its tools in Europe are condom, post-exposure prophylaxis (PEP) [6], and pre-exposure prophylaxis (PrEP) [7–10]. Secondary prevention reduces the burden of the infection (delaying or avoiding, for instance, progression to AIDS) through early detection and antiretroviral treatment. Additionally, effective ART treatment is known to prevent transmission in sero-discordant partnerships [11].

In Portugal, several national prevention programs have been targeting both the general population and key populations, including risk reduction programs for people using drugs, scale-up of PrEP, promotion of sexual and reproductive health, and prevention of violence and stigma reduction [3]. Particularly, between 2019 and 2023, non-governmental and community-based organizations, schools, and healthcare facilities distributed 25 million condoms and 6 million lubricant packages to the general population and to key populations [3]. In addition, a scale up of PrEP was possible through the decentralization and expansion of the PrEP referral network, as well as its full reimbursement through the Portuguese National Health Service. PrEP coverage is, however, still limited, averaging 7,000 users in 2023 [3]. The number of HIV tests performed – provided by primary and urgent healthcare services, as well as by community based and/or non-profit organizations [3, 4] – also increased gradually over the years, reaching 89,000 in 2023 leading the country to meet the first UNAIDS target for 2030. Stepping up the scope and effectiveness of public health programs for HIV prevention now requires an understanding of the knowledge and use of primary and secondary prevention strategies by key populations in Portugal, which include sex workers (SW), men who have sex with men (MSM), people who inject drugs (PWID), migrants and transgender people [12]. Previous research focusing on key populations in Portugal has contributed to characterize their sexual behavior, including condom use [13–23], PrEP knowledge [12], eligibility [24–27], uptake/use [28, 29], and HIV testing [30–39]. However, such studies typically focus on one prevention outcome and specific key populations. The aim of this scoping review is to contribute with observational evidence regarding the coverage, scope, and broad community impact of HIV prevention in key populations in Portugal for a better evidence-based public health action.

## Methods

### Search strategy and eligibility criteria

We conducted a scoping review on HIV prevention strategies among key populations in Portugal according to the Preferred Reporting Items for Systematic Reviews and Meta-Analyses extension for scoping reviews (PRISMA-ScR) [40] (see Table 1 in Additional file 1). This review is registered at Open Science Framework [97].

**Fig. 1:**
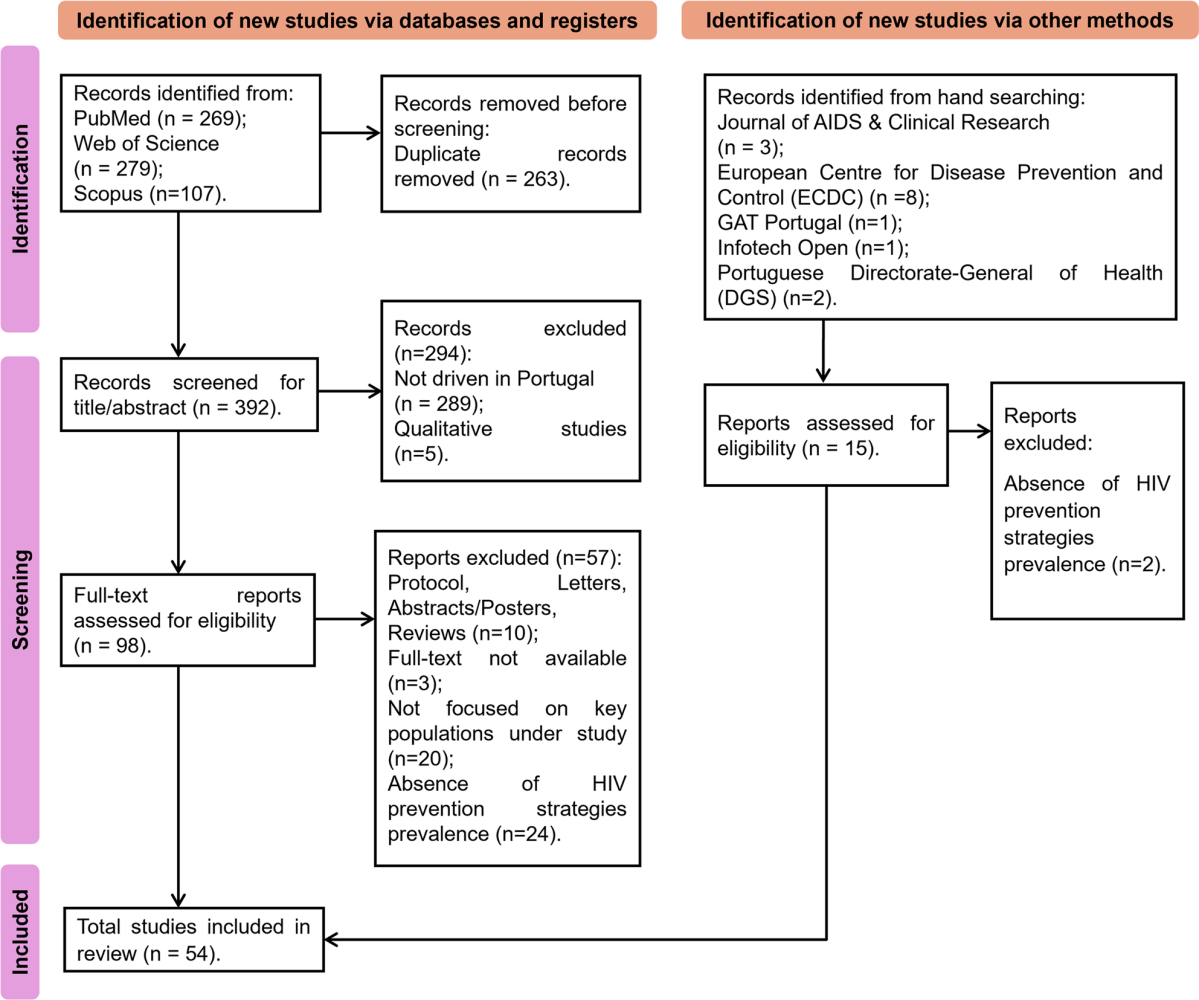
Preferred Reporting Items for Systematic Reviews and Meta-Analyses (PRISMA) flow diagram

To design the search strategy, we defined a PCC (Population, Context and Concept) framework according to Pollock et al. [41], which aimed to identify studies driven in Portugal (context), characterizing HIV primary and secondary strategies such as oral PrEP, PEP, condoms and testing (concept) within and between high-risk populations such as MSM, SW, transgender people, migrant people, and PWID (population). Article selection was restricted to studies published between 2008 and 2025, period of significant output in HIV prevention, collected from three electronic databases: PubMed, Scopus and Web of Science Core Collection (A&HCI, BKCI-SSH, BKCI-S, CCR-EXPANDED, ESCI, IC, CPCI-SSH, CPCI-S, SCI-EXPANDED, SSCI). The query search involved a Boolean combination of keywords such as “Portugal”, “MSM”, “gay”, “sex workers”, “migrants”, “transgender”, “persons who inject drugs”, “key populations”, “HIV”, “prevention”, “testing”, “screening”, “PrEP”, “PEP”, “condom” and “unprotected sex” (see Tables 1, 2, 3 and 4 in Additional file 2). The latest search was performed on August 20th, 2025. Studies included were limited to Portuguese and English languages. No other search filters were applied, and the search was conducted across all fields to ensure that no relevant research studies were excluded. All articles were transferred to Zotero, a bibliographic management system software. Duplicate articles were either manually or automatically identified by the software and removed. The article selection and screening processes were performed in a hierarchical way and split into two parts. First, records were screened by title/abstract such that those not driven in Portugal or qualitative studies were excluded. Second, articles were screened through a full-text assessment. Non-original and/or unpublished studies such as conference abstracts, study protocols, reviews and letters were excluded. For records without available full text, attempts to contact the authors were made, but no responses were obtained. Included studies focused on at least one of the previously defined key population groups and report assessment towards knowledge and/or use of at least one of the defined primary and/or secondary prevention methods. Additional records were collected through hand searching of peer-reviewed journals and/or reports published by national healthcare institutions or organizations and were screened and selected according to the previously defined eligibility criteria (see Fig. 1). To reduce the potential for reviewer bias, titles and abstracts of all identified records were independently screened by two authors (JB and BW) and checked for agreement. Subsequently, the full text of potentially relevant studies was read and independently screened for the eligibility criteria. Discrepancies in the study selection were resolved by consensus or were discussed with a third author (PM) for a final decision.

### Data charting and extraction

Data charting followed the established approach for scoping reviews. Study characteristics, such as year of publication, first author, study population size, key population under study, prevention strategies and respective assessment were extracted (see Table 1 in Additional file 3). The number of publications during the period of analysis was assessed and represented through a graph. We synthetized the instrument variables used to assess HIV primary and secondary prevention strategies, recall periods and frequency stratified by knowledge and use, and by key population, when available.

## Results

### Search and Article Selection

From the three electronic databases, a total of 655 peer-reviewed articles were identified, particularly 269 from PubMed, 279 from Web of Science, 107 from Scopus. Of those, 263 duplicate articles were identified and removed, leaving 392 articles for screening. In the first stage, 294 were excluded based on their title and/or abstract using broader criteria: first, articles that were not conducted in Portugal (*n* = 289) followed by additional qualitative studies (*n* = 5). For the second screening phase, 98 full-text articles were considered relevant for full-text eligibility assessment, from which 57 were excluded through the following hierarchical process: first, we removed non-original studies including conference posters/abstracts (*n* = 4), reviews (*n* = 2), letters (*n* = 2) and study protocols (*n* = 2); second, articles without available full text (*n* = 3); third, studies not focusing on any of the key populations under study or combined with other population groups (*n* = 20); and finally, records that did not provide estimates of uptake, usage or knowledge of any of the prevention methods (*n* = 24).

Manual searching further added articles from the Journal of AIDS & Clinical Research (*n* = 3) and from Intechopen (*n* = 1). Other literature sources considered were reports published by the European Centre for Disease Prevention and Control (ECDC) (*n* = 8), the Portuguese Directorate-General of Health (DGS) (*n* = 2), and Grupo de Ativistas em Tratamentos (GAT), a community-based organization located in Lisbon (*n* = 1). Two reports from the search did not enter in the study as they did not focus on any of the prevention strategies under study. The whole selection process resulted in the inclusion of 54 studies. See Fig. 1 for the PRISMA flowchart of the study selection and the main reasons for article exclusion in the screening process.

### General characteristics

**Fig. 2:**
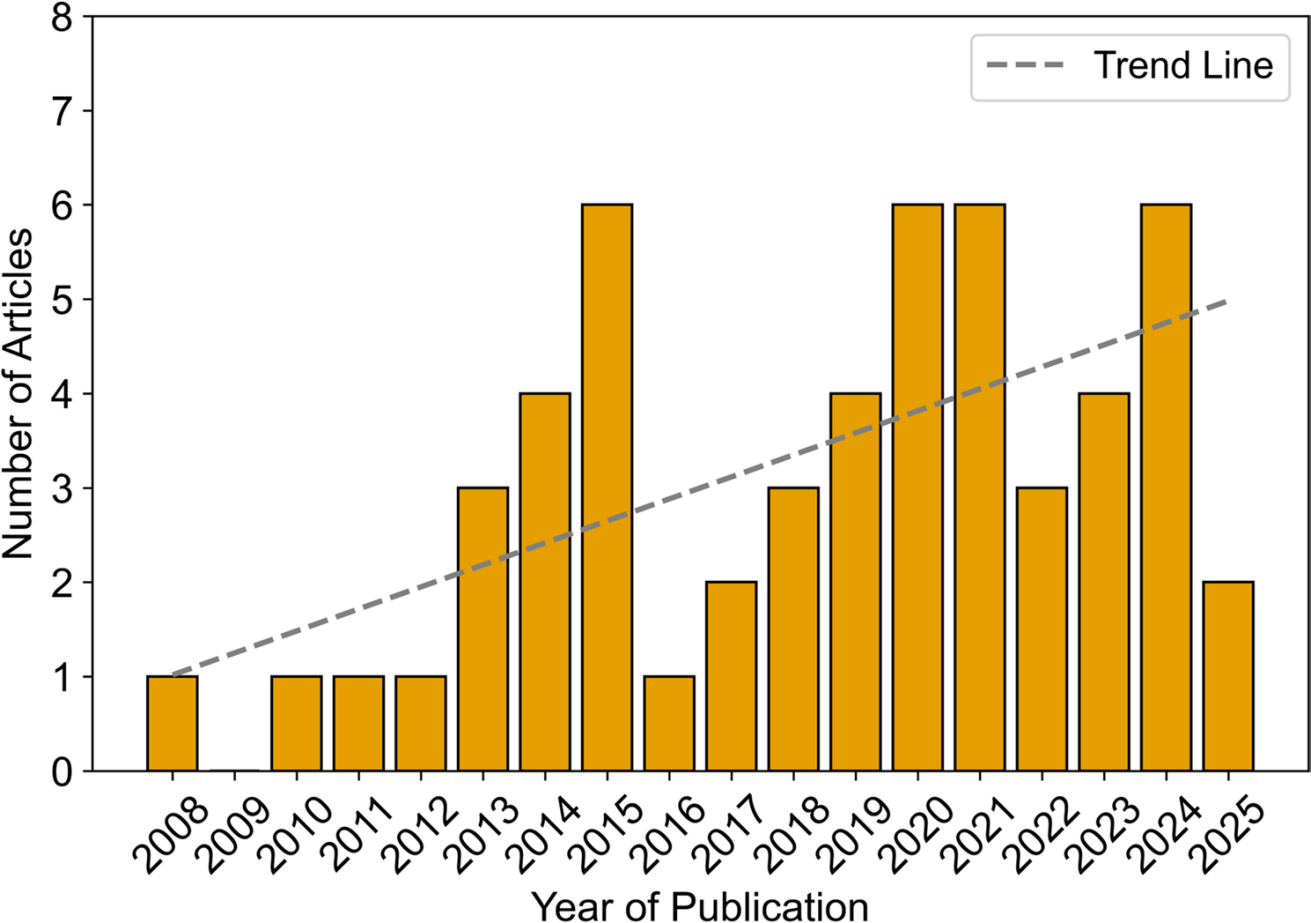
Temporal evolution of research output in HIV primary and secondary prevention strategies in high-risk populations published between 2008 and 2025

Study characteristics were collected and summarized according to year of publication, first author, study population size, key population under study, prevention strategies and respective assessment (see Table 1 in Additional file 3). The number of studies examining primary and secondary HIV prevention strategies has increased between 2008 and 2025, with over half (57.4%) published since 2019 (see Fig. 2). Most studies focused on condom usage (*n* = 44) and testing (*n* = 42). Fewer examined PrEP (*n* = 16) and PEP (*n* = 12). Some studies presented knowledge and/or used data by grouping multiple prevention strategies together. For instance, three studies reported PEP usage alongside condomless sex in the previous six months [24–27], one study reported PrEP and PEP (knowledge and usage) combined with condomless sex in the last 12 months, or ever having tested for HIV [12], and studied the combination between PrEP use and HIV testing [42].

The distribution of key populations under study varied across studies. Most studies were focused on MSM (*n* = 34) followed by migrants (*n* = 20), SW (*n* = 11), transgender (*n* = 6) and PWID (*n* = 6). Additionally, some studies examined overlapping or intersecting key population groups, such as transgender migrants [43], transgender SW [14, 20], MSM migrants [42, 44] and MSM SW [18, 44].

**Fig. 3:**
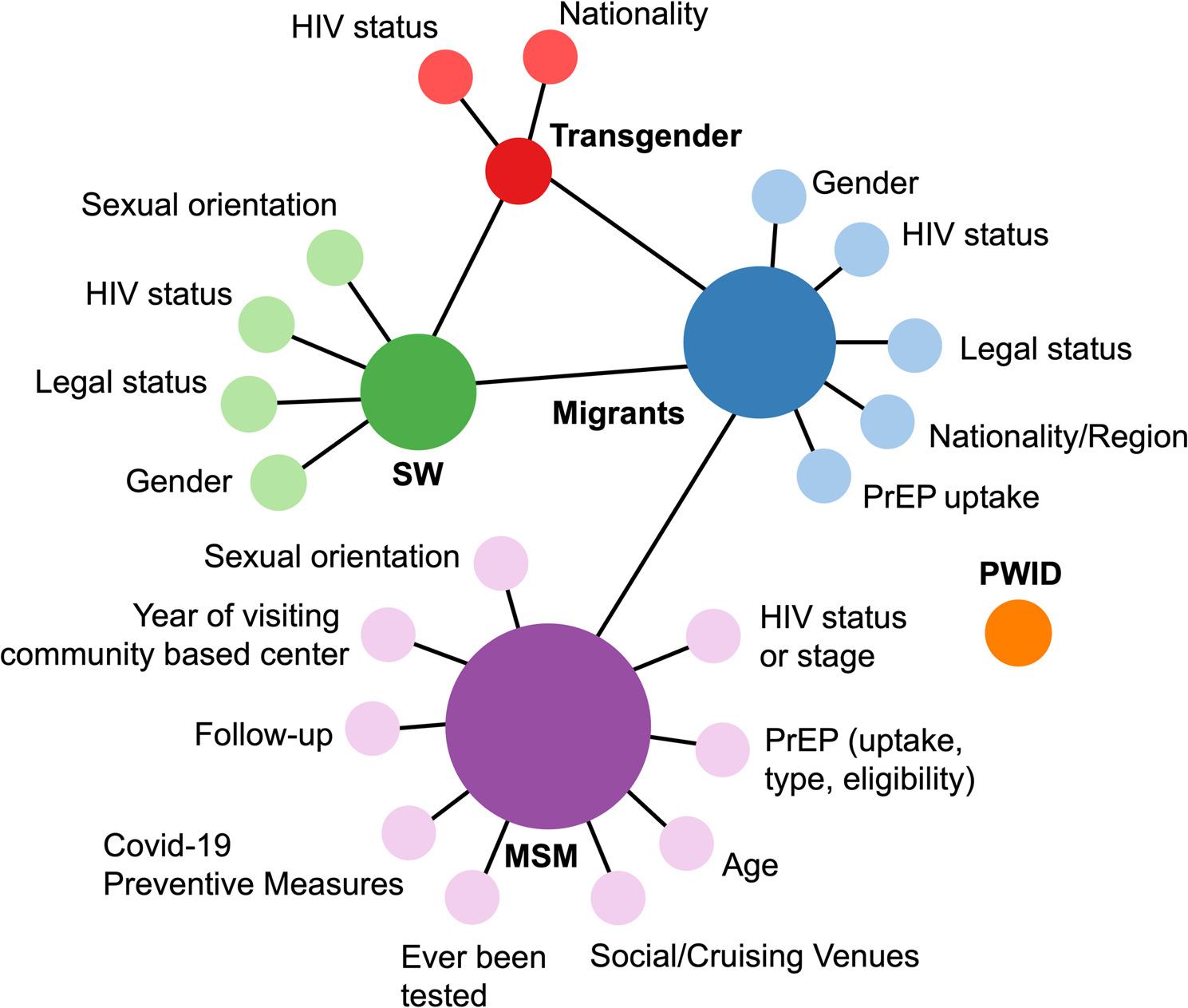
Network visualization of the overlapping and stratification of HIV prevention strategies among the key populations under study. The size of the blobs is proportional to the number of studies that characterizes each key population. The lines represent the intersection between different key populations and respective stratification in subcategories

In some studies, primary and secondary prevention strategies were also reported based on how participants were stratified or considering subcategories of a given key population. For example, knowledge and/or usage of a given prevention strategy was characterized for individuals adopting COVID-19 preventive measures [45, 46], or based on HIV status and stage [14, 31, 44, 47, 48, 69], PrEP status and uptake [26, 42], PrEP eligibility status [27], follow-up visits [27], according to the type of visited venues [13], testing status [36], gender [14, 18, 20, 47], immigration status [33], age [44, 49, 50], year of visit of a community center [44], sexual orientation [18, 29, 36, 44, 59, 60] and nationality/region [14, 23, 29, 32] (see Fig. 3). However, the level of stratification varied across key populations. For example, disaggregation of prevention assessment by sexual orientation was only found in MSM [29, 36, 44, 60] or SW [18] focused studies, and was absent in migrant, transgender, and PWID-focused studies, which limits the ability to characterize the specific prevention knowledge and use of subpopulations within these groups.

### Condom Usage

**Fig. 4:**
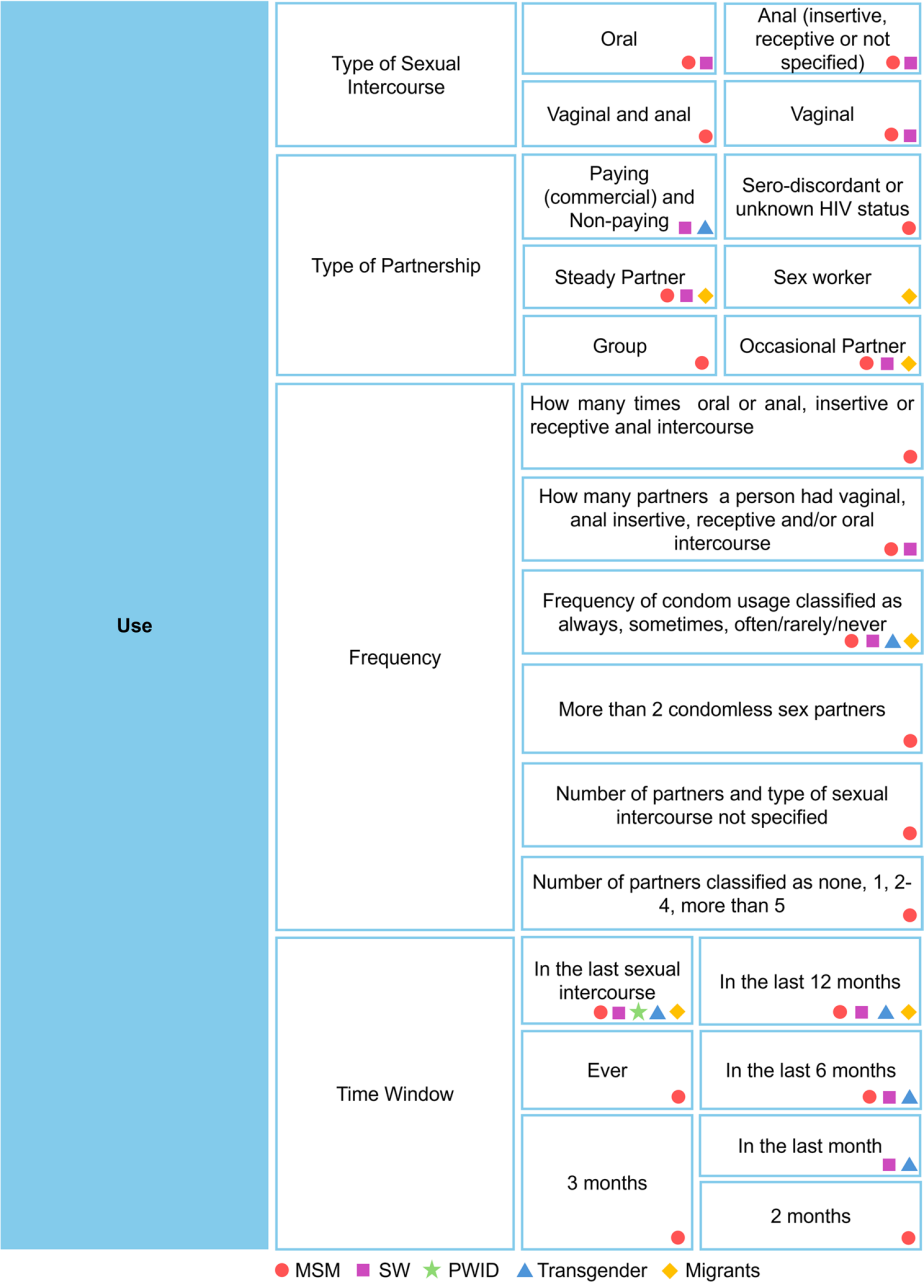
Characterization of variables to assess condom knowledge and use for all key populations. Condom use was defined according to the type of partnerships, gender, type of sexual intercourse, sexual role preference, time window, frequency. Each symbol represents a distinct key population. In cases where populations overlap, multiple symbols are shown to reflect all relevant groups

We found 44 studies that characterized condom usage, but none characterizing knowledge. Only a few studies addressed PWID and transgender people. Condom usage was defined according to the type of partnerships, gender, type of sexual intercourse, sexual role preference, time window, frequency across key populations (see Fig. 4).

Among MSM, a substantial number of studies detailed the type of condomless sexual intercourse, with many reporting on condomless anal sex [13, 15, 17, 22, 24, 27, 31, 36, 45, 49, 51–58], oral sex [15, 18, 21, 51, 59], and vaginal sex in combination (or not) with anal intercourse [21, 24, 25, 31, 42, 58, 59]. One study among male SW also reported condom usage for each type of sexual intercourse [18].

Condom usage was estimated through surveys with varying recall periods. Among MSM, condom usage was assessed based on the last time they had sex [18, 21, 22, 42, 50, 56, 60], or within a specific time window: last 2 months [51, 61], 3 months [62], 6 months [24–27, 49, 50], 12 months [12, 13, 17, 21, 36, 48, 52–58, 58] or ever [63]. The recall period was not specified in some studies. One study assessed condom usage among MSM during bathhouses visits [15] or during COVID-19-related movement restrictions [45, 46]. Among SW studies, including transgender SW, the recall periods were the last month [20], 6 months [14, 20] and 12 months [12, 14, 20, 64, 65]. For migrants, condom usage was reported according to the last time of sexual intercourse [42, 43, 60, 66, 67], and 12 months [16, 19, 47, 68, 69]. For PWID, condomless sex was only assessed at the last sexual interaction [70].

Another important metric used to characterize consistency in condom usage was its frequency, defined in multiple ways across studies. In studies on migrants, MSM and SW, some used Likert-type scales [71] to assess general condom usage trends [13–15, 18–21, 47, 57, 59, 62, 68, 69].

Among MSM, some reported the number of condomless sexual partnerships [15, 49, 51, 53–56, 61], while others also characterized condom use frequency according to sexual role preference, type of sexual intercourse, and/or number of condomless acts with men [15, 24, 51, 61].

Condom consistency was also characterized by partnership type (steady/regular, non-steady, occasional) for migrants, MSM and SW [13, 16, 17, 21, 24, 47, 49, 52, 56, 64, 72]. Among MSM, group sex [13, 48, 58], sex in serodiscordant partnerships [13, 17, 24, 52] and sex with partners with unknown HIV status [13, 21, 22, 24–27, 36, 56, 62] were also reported. One study, which focused on migrants, reported condomless sex with SW [47]. Among SW, including transgender SW, condom usage was also studied across paying/commercial, non-paying partners and group sex [14, 64, 65].

Finally, condom breakage and slippage were reported for MSM [17, 21], male, female and transgender SW [14, 20].

### PrEP and PEP

**Fig. 5:**
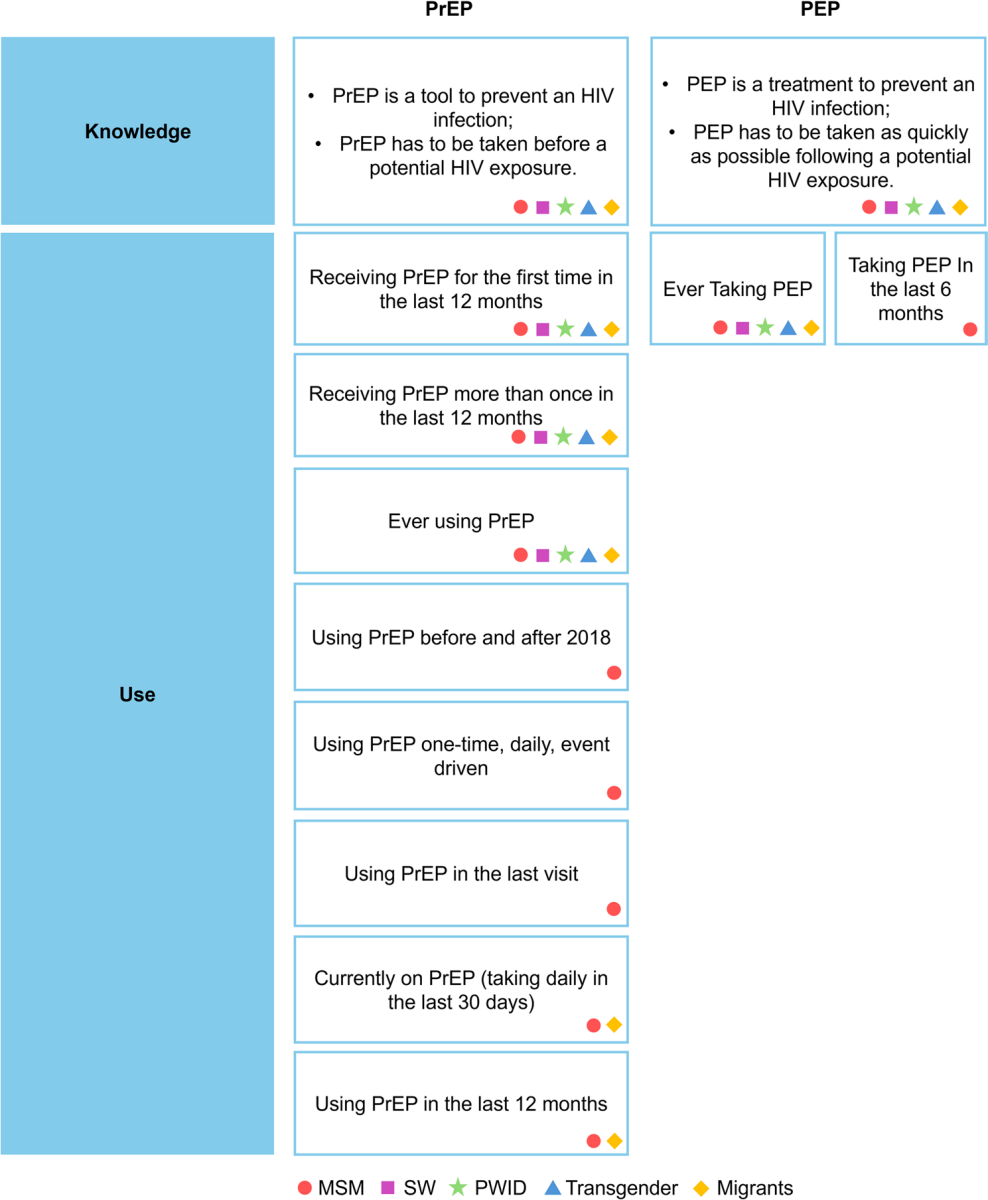
Characterization of variables used to assess PrEP and PEP knowledge and use across all key populations. Each symbol represents a distinct key population. In cases where populations overlap, multiple symbols are shown to reflect all relevant groups

From the selected articles, we identified 16 studies focused on PrEP and 12 on PEP. Knowledge and usage were evaluated using multiple assessment metrics (see Fig. 5).

One study used a Likert-type scale by asking participants to measure their understanding of each prophylactic strategy. Particularly, they assessed participant ability to recognize PEP as post-exposure prophylaxis that can be initiated as quickly as possible after exposure or how it should be taken [12, 52, 56], and to recognize PrEP as prophylaxis to be taken before exposure and regime [12, 56]. One study assessed PrEP and PEP knowledge but does not specify how it was assessed [57]. Regarding usage, one study characterized both PrEP and PEP usage proportional to each key population [12].

Some studies focused exclusively on PrEP, reporting the number or proportion of participants who initiated and/or took PrEP in the previous 12 months [3, 4, 29, 42, 44, 57, 60], as well as those who had ever received PrEP [3, 4, 73] or the proportion of PrEP users at the time of first visit [44]. However, some studies reported only the proportion of key populations among PrEP users, without providing the proportion within each group [3, 4, 65, 73]. No studies were found characterizing knowledge and/or usage of injectable PrEP.

A broader set of assessment indicators was found in MSM-focused studies. Current PrEP status was characterized (defined as taking a daily PrEP tablet in the last 30 days) [29, 74, 75], as well as prior PrEP experience, and the type of PrEP regimen used (daily, on-demand, or single-use) [29, 57]. One focused on the interaction between PrEP use and COVID-19-related nonpharmaceutical interventions [46]. Some studies also distinguished PrEP usage before and after 2018 [29], the year when Portugal started to deliver PrEP for HIV prevention, fully reimbursed [76, 77]. Two MSM focused studies also assessed if participants have ever taken PrEP [25, 45]. Regarding PEP usage, the typical reported period was within the last 6 months [24, 26].

### Testing

**Fig. 6:**
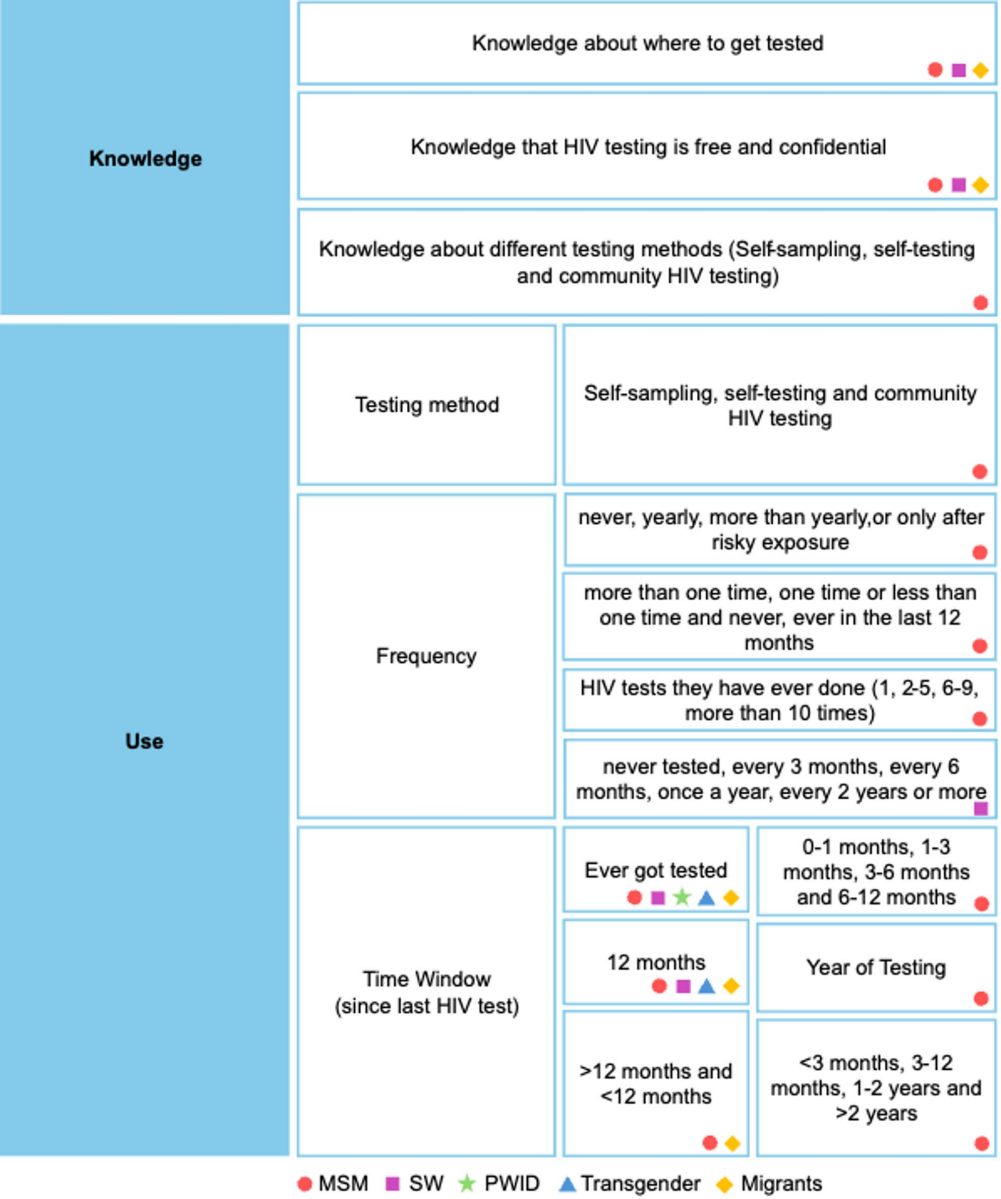
Characterization of variables used to assess HIV testing knowledge and use across all key populations. HIV testing was defined for different recall periods and frequency scales. Each symbol represents a distinct key population. In cases where populations overlap, multiple symbols are shown to reflect all relevant groups

We identified 42 studies that characterized HIV testing knowledge and use across key populations (see Fig. 6). Knowledge was defined across studies as being aware of where to get tested and/or the fact that HIV testing is confidential and free [33, 52]. Two studies focused on MSM and characterized both knowledge and use of HIV testing using other different testing modalities such as self-testing kits, self-sampling kits, and community-based testing [53, 56].

The most reported indicator was the proportion of participants who have ever been tested for HIV, found in 18 articles from the available literature and for all key populations, including MSM [13, 21, 24, 25, 36, 59, 61], PWID [70], female, male and transgender SW [14, 20, 33, 64], and migrants [16, 19, 23, 32, 33, 37, 67, 68]. Testing in the last 12 months was also frequently reported across MSM [17, 36, 42, 46, 49, 50, 52, 56, 60], female, male and transgender SW [14, 18, 20, 64, 65], migrants [16, 23, 42, 60] and PWID [78]. One study focused on migrants also reported the number of participants who got tested in the last 12 months [43] rather than the proportion. The testing frequency was also assessed, mainly through a Likert-type scale. Within the most reported recall period, MSM focused studies reported HIV testing frequency (e.g., more than once, once, less than once, never) [48] or ever (never, yearly, more than yearly, or only after risky exposure) [58, 62], and the number of lifetime tests [21, 54, 55, 61]. Studies on SW also reported frequency of testing (e.g., every 3 or 6 months, yearly, every two years or more) [18]. Different recall periods to survey HIV testing were used, presenting the year participants got tested [49] or the time since last test: : 0–1 months, 1–3 months, 3–6 months and 6–12 months [26], more than 12 months ago or less than 12 months ago [53] or < 3 months, 3–12 months, 1–2 years and > 2 years [54, 55]. For studies exclusively focused on migrants, additional assessment instruments included the time since the HIV test was performed (less than a year or more than a year) [68].

## Discussion

This work provides a comprehensive overview of studies published between 2008 and 2025 estimating and investigating the use, uptake and knowledge of primary and secondary prevention strategies of HIV among key populations in Portugal. Our findings highlight an increase in research output over time, with more than half of the studies published between 2019 and 2025. This trend has been keeping up with improvements in the current national HIV prevention programs focused on the promotion of tailored interventions among key populations by increasing the availability and coverage of HIV testing tools, PrEP and condoms [4]. While such efforts have contributed to a 37% reduction in HIV acquisitions between 2014 and 2023, HIV incidence in Portugal has remained relatively stable in recent years, while other European Union countries have achieved lower rates, which suggests the need to rethink current HIV prevention strategies implementation in Portugal. For example, despite the scale-up of PrEP through the decentralization and expansion of the PrEP referral network as well as its full reimbursement through the Portuguese National Health Service [3], PrEP coverage was still limited to 7000 users until 2023, with the proportion of PrEP users within key populations falling below that of other European countries [73]. Given that knowledge and use of prevention strategies are key indicators for evaluating the reach and effectiveness of prevention programs, we summarized evidence to map how these indicators are assessed. However, we identified important knowledge gaps, mostly related to the lack of characterization and high heterogeneity in the assessment of HIV prevention methods within and across key populations. These gaps identify areas where future investigation is needed and as discussed below, currently limit the ability to evaluate which interventions are most effectively reaching key populations and where policy efforts should be strengthened.

Most studies included in this scoping review were focused on HIV testing and condoms, while a lower amount of literature was found for PrEP and PEP. PrEP was authorized in the EU through a recommendation by the European Medicines Agency in 2016 [79]. In February 2018, Portugal started to provide and fully reimburse PrEP through the nation healthcare service [76, 77]. PEP, while being available before PrEP, is still limited in its access, largely being reserved to occupational exposures among healthcare workers [80]. This might explain the smaller amount of literature covering PEP. Despite the limited available literature, some studies combine PEP or PrEP knowledge and usage with other prevention strategies. Specifically, PEP and/or PrEP usage was reported alongside with condom usage in the past 6 [26, 27] and 12 months [12], making it difficult to disentangle the adoption and impact of each prevention method and, consequently, to compare them across key populations. Another relevant knowledge gap is the lack of studies assessing knowledge and usage of injectable PrEP. Currently, two types of injectable PrEP are authorized in the EU: cabotegravir since September 2023 [81], and lenacapavir, very recently, since August 2025 [82]. Since injectable PrEP has proven to be more efficient than oral PrEP, further studies are required to understand whether this new prevention tool is reaching high risk populations. The recent availability of injectable PrEP and the fact that it is currently not provided by the national healthcare service of Portugal obviously explain the knowledge gap in the literature. Notwithstanding, the high effectiveness and reduced adherence burden of injectable PrEP make it a cornerstone of future primary prevention of HIV [83, 84]. Studies regarding the acceptability and effectiveness of injectable PrEP among key populations in Portugal are urgently needed to inform future public health policies and cost-effectiveness analyses.

Our scoping review highlights the uneven distribution of studies across key populations, entailing substantial knowledge gaps. Specifically, SW and PWID were underrepresented, and no study focused exclusively on transgender people. MSM have been the focus of active research through various research projects such as the Lisbon Cohort [17, 21, 24, 25, 27, 29, 57], EURO HIV EDAT [53–55], Project PREVIH [22], In PrEP [42, 45], Sialon II [49, 50], EMIS-17 [56] and EMIS 2010 [36, 52], which explains the broader and richer knowledge and use prevention strategies characterization within this key population. Other key populations vulnerable to HIV have instead been covered less effectively. This is also true in the case of overlapping vulnerabilities, such as transgender SW and migrant SW [85]. Their experience of intersectional stigma may constitute a barrier to HIV detection and evidence-based preventative policies [86, 87]. Moreover, alongside expanding knowledge in intersectional communities, our work identified that the level of stratification varied across key populations. In migrant, transgender and PWID focused studies prevention strategies assessment did not disaggregate prevention assessment by sexual orientation, which limits the ability to characterize the specific prevention needs of subpopulations within these groups, such as heterosexual and homosexual migrants.

The assessment of knowledge and usage within primary prevention strategies was also unequal. Specifically, no studies were conducted to characterize knowledge about condom. Without such baseline knowledge surveillance data, it is not possible to determine whether awareness campaigns are needed or whether existing efforts are reaching key populations effectively. To strengthen structural prevention efforts aimed at reducing HIV incidence among key populations, further studies could incorporate knowledge assessment by asking participants if condoms are the best way of protecting against HIV [88], and about the appropriate conditions for condom use, such as timing and correct usage techniques [89].

A high methodological heterogeneity was found across studies in the assessment of primary and secondary prevention strategies. First, HIV testing and condom usage were assessed for different frequency ranges and timeframes. Second, metrics assessment for the same prevention strategy outcome was also inconsistent. Particularly, while some studies reported the assessment outcome as a proportion, others only present the number of participants or the proportion of each key population group that uses and/or knows a given prevention strategy [3, 4]. This heterogeneity within and across instruments assessment, was also reported by the mapping of HIV/STI behavioral surveillance in Europe report [90, 91] which might be explained by the fact that studies were conducted in different recruitment places, target populations, study type, objectives, lack of resources and/or cost. However, in order to design and propose more targeted and tailored interventions to high risk populations, surveys on behavioral data for HIV prevention and control need to be robust and comparable [91]. The need for standardized metrics of knowledge, uptake and behavioral effect of prevention strategies is important to develop modeling studies that can help evaluate and propose public health policies, as it will be necessary, for instance, for injectable PrEP. This can partially explain a methodological knowledge gap that we identified: the absence of modeling studies that evaluate the effectiveness and cost-effectiveness of HIV prevention in Portugal and will be crucial in helping guide future public health policies, such as the introduction of injectable PrEP. The lack of harmonized and comparable data across studies also restricts the capability of mathematical models to capture, for example, transmission pathways across key populations, which potentially exist [47, 92]. Consequently, if models simplify population structure by treating key populations as isolated and homogeneous groups, overlooking the complex network of interactions that more realistically drives HIV transmission, they will underestimate transmission and bias policy evaluation. In order to harmonize the comparability of different indicators, a possible solution would be to drive further studies following the recommended guidelines proposed in the Global AIDS Monitoring 2025 designed to enable the best use of available data at the national level, to standardize reporting from different HIV epidemics and sociopolitical contexts [93].

Our work has, however, some limitations. First, in our article search we compared articles that represent populations at different moments in time, which can explain why we detected a significant amount of heterogeneity across studies and key populations. Second, searching across All Fields is literal and does not cover full text, which means studies using terminology not included in our query may not have been captured. However, we supplemented database searching with a thorough hand searching of reports and journals from public health and government institutions, to mitigate this constraint and minimize the risk of missing relevant studies. Third, the characterization of the primary and secondary prevention strategies of each key population based on knowledge and usage might have excluded important assessment indicators. These include adherence to prevention methods and follow up patterns [94–96] which are crucial for understanding the sustained effectiveness of interventions. Furthermore, the exclusion of the willingness to use prevention strategies may have overlooked important data on potential uptake of existing methods. The assessment of HIV transmission-related knowledge, accessibility, perception of prevention methods [52, 56], and respective coverage [14, 16, 33] were also not captured in our review framework, also important in characterizing the effectiveness of current healthcare programs within and across key populations.

## Conclusions

This scoping review examined the assessment of primary and secondary prevention of HIV among key populations in Portugal. Our review identified important knowledge gaps in selected key populations, notably transgender and PWID, and suggested areas where immediate research is needed, such as the acceptability and effectiveness of injectable PrEP. Additionally, prevention uptake was better studied than knowledge of prevention among all key populations. We also found a high level of heterogeneity in methodological approaches to assess prevention outcomes. This highlights the importance of driving further studies to develop more standardized tools to improve knowledge and use of primary and secondary HIV prevention assessment metrics comparison between and across key populations and to drive mathematical modelling studies to improve the efficiency of the current implemented HIV prevention programs. Our contribution highlights that improving the comparability of metrics assessments is a necessary step toward identifying which key populations are being left behind and to propose more tailored prevention strategies to such populations. Without reliable and comparable data across populations, healthcare policy makers ability’ to evaluate the impact of existing prevention programs and to introduce new technologies such injectable PrEP remains limited.

## Supplementary Information

Supplementary Material 1.

Supplementary Material 2.

Supplementary Material 3.

## Data Availability

Data sharing is not applicable to this article as no datasets were generated or analyzed during the current study.

## Abbreviations

HIV: Human Immunodeficiency Virus
AIDS: Acquired Immunodeficiency Syndrome
MSM: Men Who Have Sex With Men
SW: Sex Workers
PWID: People Who Inject Drugs
ART: Antiretroviral Therapy
ECDC: European Centre for Disease Prevention and Control
PrEP: Pre-exposure prophylaxis
PEP: Post-exposure Prophylaxis
UNAIDS: Joint United Nations Programme on HIV/AIDS
PRISMA-ScR: Preferred Reporting Items for Systematic reviews and Meta-Analyses extension for Scoping Reviews
PCC: Population, Context, Concept
DGS: Portuguese Directorate-General of Health
GAT: Grupo de Ativistas em Tratamentos
