# Supplementary material for: Assessment of knowledge and use of HIV primary and secondary prevention strategies in Portugal: a scoping review
Source: BMC Public Health. 2026 May 7;26:1964. doi: 10.1186/s12889-026-27542-7 (PMC13317048; doi:10.1186/s12889-026-27542-7)
Supplement: Supplementary file 1 — Supplementary Material 1. [file 12889_2026_27542_MOESM1_ESM.docx]

**Assessment of Knowledge and Use of HIV Primary and Secondary Prevention Strategies in Portugal: A Scoping Review**

João Brázia^1,2, §,^ Boxuan Wang^3^, Paula Meireles^4^, Eugenio Valdano^3^, Andreia Sofia Teixeira^1,2^

1. BRAN Lab, Network Science Institute, Northeastern University London, London, UK.
2. LASIGE, Faculdade de Ciências, Universidade de Lisboa, Portugal.
3. Sorbonne Université, INSERM, Institut Pierre Louis d’Epidémiologie et de Santé Publique, F75012, Paris, France.
4. EPIUnit ITR, Instituto de Saúde Pública da Universidade do Porto, Universidade do Porto, Rua das Taipas, n° 135, Porto 4050-600, Portugal.

^§^ Corresponding author: João Brázia, Devon House, 58 St Katharine's Way, London E1W 1LP, [simoesbrazia.j@northeastern.edu](mailto:simoesbrazia.j@northeastern.edu)

**Additional file 3**

This file presents the data charting followed the established approach for scoping reviews. Study characteristics, such as year of publication, first author, study population size, key population under study, prevention strategies and respective assessment were extracted (Table 1).

Table 1 Synthesis of included studies in the scoping review

| Year of Publication | Author | Sample size (n)/key population | Prevention Strategies | Outcome assessment |
| --- | --- | --- | --- | --- |
|  |  |  |  |  |
| 2008 | Margarida Gaspar de Matos et al. [66] | 743 migrants | Condom | ● **Condom:** proportion of participants who condom use in the last sexual intercourse; |
| 2010 | Ana Gama, et al. [37] | 522 Migrants | HIV testing | ● **Testing:** Proportion of participants who have ever got tested for HIV; |
| 2011 | Sónia Dias, et al. [32] | 1513 Migrants | HIV testing | ● **Testing:** Proportion of participants who have ever got tested for HIV; who had their last test in the previous 4 years; |
| 2012 | Sónia Dias, et al. [23] | 1282 Migrants | HIV testing | ● **Testing:** Proportion of participants who have ever got tested for HIV; who ever got tested for HIV in the last 12 months. |
| 2013 | Celeste Carvalho, et al. [36] | 5187 MSM | HIV testing and condom | ● **Testing:** Proportion of participants who have ever got tested for HIV; who ever got tested for HIV in the last 12 months (of those without known infection);  ● **Condoms:** Proportion of participants who reported CAI with a partner of unknown or Sero discordant status in the previous 12 months (among those who never tested); that reported CAI in the last 12 months (stratified by have or have never been tested). |
| 2013 | ECDC [78] | PWID (size not provided) | HIV testing | ● **Testing:** Proportions of participants reporting having received an HIV test within the last 12 months and who also knew the result of that test |
| 2013 | ECDC [52] | 5406 MSM | HIV testing, Condom and PEP | ● **Testing:** proportion of participants who have been tested for HIV in the last 12 months; HIV testing knowledge (AIDS is caused by a virus called HIV; there is a medical test that can show whether or not you  have HIV; someone becomes infected with HIV it may take several weeks before it can be detected in a test, there is currently no cure for HIV infection; HIV infection can be controlled with medicines so that its impact on health is much less).  ● **Condom:** proportion of participants who reported CAI during last episode of sex abroad; with steady and non-steady partner and/or male partner (including unknown, HIV-discordant or non-concordant) in the last 12 months; with last non-steady partner in the last 12 months.  ● **PEP:** Proportion of participants who reported PEP knowledge (PEP attempts to stop HIV infection from occurring after a person is exposed to the virus; is a one-month course of anti-HIV drugs; should be started as soon as possible after exposure, preferably within hours) and used PEP; |
| 2014 | Katie B. Biello, et al. [62] | 4111 MSM | HIV testing and condom | ● **Testing:** proportion of participants HIV testing frequency (classified as never, yearly, more than yearly, or only after risky exposure); ● **Condoms:** proportion of participants who had any unprotected intercourse with a partner of different or unknown HIV sero-status in the last 3 months; condom use frequency in the past 3 months (never, inconsistent, and always) |
| 2014 | Henrique Pereira [51] | 304 MSM | Condom | ● **Condom:** number of sexual acts with the same partner and number of men with which participants had insertive/receptive anal/oral sex in condomless sex in the last 2 months; |
| 2014 | Sónia Dias, et al. [16] | 1187 Migrants | HIV testing and condom | ● **Testing:** Proportion of participants who have ever got tested for HIV; who ever got tested for HIV in the last 12 months; ● **Condom:** proportion of participants who reported condom in last sexual encounter; who reported condom consistency (those who always used condoms) with occasional and regular partners in the last 12 months;  ● Prevalence stratified by gender. |
| 2014 | Henrique Pereira, et al. [18] | 143 SW | HIV testing and condom | ● **Testing:** proportion of participants who ever got tested for HIV in the last 12 months; HIV screening according to frequency (never tested, every 3 months, every 6 months, once a year, every 2 years or more). ● **Condom:** frequency of condomless sex in oral, anal or vaginal sex (rarely, always, almost always and doesn’t apply); proportion of condom usage in the last sexual relationship with a partner (anal or in general);  . |
| 2015 | Paula Meireles, et al. [17] | 804 MSM | HIV testing, condom and PEP | ● **Testing:** Proportion of participants who ever got tested for HIV in the last 12 months;  ● **Condom:** proportion of participants who reported condom rupture; CAI with occasional partner,HIV positive steady or steady partner in the last 12 months  ● **PEP:** proportion of participants who reported knowledge and usage; |
| 2015 | Sónia Dias, et al. [20] | 1040 SW and transgender | HIV testing and condom | ● **Testing:** Proportion of participants who have been tested for HIV or in the last 12 months.  ● **Condom:** proportion of non-consistent condom use with non-paying partners in the last 12 months; consistent and non-consistent (sometimes/never) condom use with clients in the last month; frequency condom failure in the last 6 months.  ● Prevalence stratified by gender (male, female and transgender). |
| 2015 | Paula Meireles, et al. [21] | 2183 MSM | HIV testing, condom and PEP | ● **Testing:** Proportion of participants who have ever got tested for HIV; number of previous HIV tests;  ● **Condom:** frequency of condom use in the last 12 months with steady partner of unknown HIV status, occasional partner or HIV-positive steady partner (always, often/occasionally/rarely/ever,rather not to say); proportion of condom use with steady or occasional partner in the last sexual encounter; frequency condom use in anal or oral intercourse (always, often/rarely/never and rather not to say); proportion of participants which reported condom rupture or left inside.  ● **PEP:** knowledge and use. |
| 2015 | Nuno Nodin, et al. [59] | 393 MSM | HIV Testing and condom | ● Testing: Proportion of participants who have ever got tested for HIV; ● Participants were asked when the last time was they get tested for HIV but prevalence not available; **● Condom:** condom usage assessed through Likert scale (e.g., 1 = "Never", 5 = "Always"), and the mean gives an idea of the general trend among each group; three types of condom usage, for vaginal, anal and oral sex; |
| 2015 | Henrique Pereira et al. [61] | 304 MSM | HIV testing and condom | ● **Testing:** proportion of knowledge of where to get tested; number of times got tested for HIV; and proportion of participants who ever got tested;  **● Condom:** frequency of condomless sex in the last 2 months regarding receptive/insertive anal/oral sex (how many men/ how many times); |
| 2015 | Samuel Monteiro, et al. [15] | 424 MSM | Condom | ● **Condom:** frequency in oral/anal receptive/insertive condomless sex expressed in likert-scale (1=never; 5=always); frequency and proportion of participants that reported ejaculation in anal/oral receptive/insertive condomless sex; |
| 2016 | Massimo Mirandola, et al. [49] | 408 MSM | HIV testing and condom | ● **Testing:** proportion of participants according to year they got tested for HIV (<2012, >2012 and never tested) stratified by age (<25 or +25); knowledge about where to get tested aggregated with in the last 12 months; having received an HIV test within the last 12 months and who also knew the result of that test;  ● **Condoms:** proportion having had an unprotected intercourse (steady and non -steady, non-steady or steady partner, no partner) in the last six months; who reported condomless sex according to the number of partners (no partner, 1 partner, 2-3 partners, 4-5 partners, >5 partners) in the last six months; sexual intercourse with a female partner (steady and non-steady) in the last six months; condom use during last anal intercourse with a male partner stratified based on partner type (steady or non-steady more than one partner) or sexual role (insertive, versatile and receptive) or age (more than 25, less than 25 and total for steady, non-steady and in general); |
| 2017 | Ana Gama, et al. [13] | 1046 MSM | HIV testing and condom | ● **Testing:** Proportion of participants who have ever got tested for HIV.  ● **Condom:** Proportion of participants who revealed unprotected anal sex with regular partners, occasional partners, in group sex in the last 12 months (always, sometimes/rarely/never); who engaged in UAI with partner with unknown or sero discordant HIV serostatus in the last 12 months; ● Prevalence given in general but also stratified according to attendance of participants to cruising or social gay venues. |
| 2017 | Sónia Dias, et al. [33] | 784 Migrants and SW | HIV testing | **Testing:** proportion of participants who have ever got tested for HIV; knowledge that HIV testing is free and confidential or where to get tested; ● Participants testing prevalence reported in general and also stratified based on legal and migration status (documented, undocumented migrants and national). |
| 2018 | Ana Gama, et al. [14] | 125 Transgender and SW | HIV testing and condom | **Testing:** Proportion of participants who have been tested for HIV.  **Condom:** Proportion of non-consistent condom use (sometimes/never) with non-paying partners in the last 12 months or with clients in the last month; reporting condom failure in the last 6 months. |
| 2018 | Juan Hoyos, et al. [53] | 755 MSM | HIV testing and condom | ●**Testing:** proportion of participants who reported knowledge about self-sampling kits and their usage, who ever got tested for HIV, tested more than 12 months ago and tested less than 12 months ago;  ● **Condom:** proportion of participants according to number of partners with CAI in the last 12 months (none, 1, 2-4, more than 5). |
| 2018 | Massimo Mirandola, et al. [50] | 408 MSM | HIV testing and condom | ● **Testing:** proportion of participants reporting having received an HIV test within the last 12 months and who also knew the result of that test.  ● **Condom:** Condom usage defined as the ratio between the number of MSM who reported that a condom was used the last time they had anal sex by the number of MSM who reported having had anal sex with a male partner in the last 6 months. |
| 2019 | Sofia Ribeiro, et al. [26] | 444 MSM | HIV testing, condom and PEP | ● **Testing:** proportion of participants according to time they got tested last time (0-1 months, 1-3 months, 3-6 months and 6-12 months);  ● **Condom and PEP:** proportion of people who had condomless sex in the past 6 months and sexual partners with unknown HIV status; who have had condomless sex in the past 6 months and used PEP for HIV; |
| 2019 | ECDC [56] | 2555 MSM | HIV testing, Condom, PEP and PrEP | **Testing:** Proportion of participants who ever got tested for HIV in the last 12 months; have used community HIV testing in the last HIV test; have used self-sampling or self-sampling kits in the last HIV test; not knowing where to get tested among those never tested; knowledge assessment - including that knowing there is a medical test that can show whether or not you have HIV.  **Condom:** Proportion of participants with more than 2 condomless steady partners in the last 12 months; condomless anal intercourse with non-steady partners of unknown HIV status, in the last 12 months; inconsistent condom use for anal intercourse, last encounter;  **PEP:** Proportion of participants who took PEP ever, excluding HIV diagnosed men; knowledge about PEP - it attempts to stop HIV infection taking place after a person is exposed to the virus, such as having intercourse without a condom, is a one-month course of anti-HIV drugs, should be started as soon as possible after exposure, preferably within hours;  **PrEP:** Proportion of participants currently taking PrEP excluding undiagnosed men; using U=U or PrEP for prevention of HIV transmission, in last encounter, excluding men without AI; knowledge about PrEP - that involves someone who does not have HIV taking pills before as well as after sex to prevent them getting HIV; can be be taken as a single daily pill if someone does not know in advance when they will have sex; and that if someone knows in advance when they will have sex, PrEP needs to be taken as a double dose approximately 24 hours before sex and then at both 24 and 48 hours after the double dose. |
| 2019 | Sónia Dias, et al. [69] | 790 Migrants | HIV testing and condom | **Testing:** proportion of participants who have been tested.  **Condom:** proportion of participants who used condoms in the last 12 months (always/sometimes/never); |
| 2019 | Sofia Ribeiro, et al. [63] | 1832 MSM | condom | **Condom:** proportion of participants who reported consistent condom use in all sexual practices; |
| 2020 | Paula Meireles, et al. [24] | 3713 MSM | HIV testing, PEP and condom | **Testing:** Proportion of participants who have ever got tested for HIV;  **Condom:** Proportion of participants had condomless sex in the past 6 months and sexual partners with unknown HIV status; any anal sex without condoms (receptive or insertive) in the past 6 months; inconsistent condom use with casual partners or with HIV positive partners who are not receiving treatment; vaginal or anal sexual intercourse without a condom with more than one partner.  **PEP:** Proportion of participants who used PEP and/or exposure in the past 6 months.  **Condom and PEP:** proportion of participants who have had condomless sex in the past 6 months and used PEP for HIV; |
| 2020 | Paula Meireles, et al. [25] | 3713 MSM | HIV testing, PrEP, PEP and condoms | ● **Testing:** Proportion of participants who have ever got tested for HIV;  ● **Condom:** Proportion of people who engaged in condomless sex in anal or vaginal sexual intercourse with more than 1 partner; any condomless anal (receptive insertive) in the past 6 months; condomless sex with casual partners or people who had condomless sex in the past 6 months and/or sexual partners with unknown HIV status.  ● **PEP:** Proportion of people who used PEP for sexual exposure in the past 6 months;  ● **PrEP:** Proportion of people who had used PrEP before.  ● **Condom and PEP:** proportion of participants who used PEP and had condomless sex in the last 6 months; |
| 2020 | Sónia Dias, et al. [19] | 790 Migrants | HIV testing and condom | **●Testing:** Proportion of participants who have ever got tested for HIV; ● **Condom:** Proportion of participants who reported condom use in the last 12 months (always, sometimes or never); |
| 2020 | Tomás Maté, et al. [54] | 531 MSM | HIV testing and condom | **● Testing:** Proportion of participants according to recall period of last HIV test (<3 months, 3-12 months, 1-2 years and >2 years) and number of HIV tests they have ever done (1, 2-5, 6-9, more than 10 times);  ● **Condom:** frequency of CAI according to number of partners in the last 12 months (none, 1, 2-4, more than 5) |
| 2020 | Nilza Almeida, et al. [64] | 100 SW | HIV testing and condom | ● **Testing:** Proportion of participants who have ever got tested for HIV or in the last 12 months; ● **Condom:** Proportion of condom usage in the last 12 months with regular, occasional or commercial partner. |
| 2020 | Sónia Dias, et al. [67] | 790 Migrants | HIV testing and condom | ● **Testing:** proportion of participants who reported ever having an HIV test; ● **Condom:** proportion of participants reporting condom usage with last partner (regular or casual).. |
| 2021 | Daniel Simões, et al. [12] | 2,277 MSM, 456 PWID, 2,373 SW, 9392 migrants and 148 transgender | All prevention methods | ● **Testing:** Proportion of participants who have ever got tested for HIV (aggregated for all key populations);  ● **Condom:** proportion of condom usage in the last 12 months (aggregated for all key populations);  ● **PrEP:** proportion of knowledge and use knowledge of PrEP - prevents HIV infection and needs to be taken before HIV exposure – accessed as inappropriate, incomplete or correct;  ● **PEP:** proportion of knowledge and use; knowledge of PEP - as a treatment to prevent HIV infection and be taken as quickly as possible – accessed as inappropriate, incomplete or correct. |
| 2021 | Juan Hoyos, et al. [55] | 417 MSM | HIV testing and condom | ● **Testing:** proportion of participants according to number of HIV tests they have ever done (1, 2-5, 6-9, more than 10 times) and according to time of last HIV test (<3 months, 3-12 months, 1-2 years and >2 years) for baseline and those who report that prefer self-testing; ● **Condom:** frequency of CAI according to number of partners (in the last 12 months); |
| 2021 | Paula Meireles, et al. [29] | 6164 MSM | PrEP and PEP | **PEP:** proportion of participants knowledge  **PrEP:** Proportion of participants who took PrEP in the last 12 months or in the last recent visit; PrEP usage regime (one-time, daily or event-driven) or before and after 2018; |
| 2021 | Karel Blondeel, et al. [22] | 1046 MSM | condom | ● **Condom:** Proportion of CAI among participants in the last 12 months with partner of unknown HIV status or condom use in last AI; |
| 2021 | Álvaro Francisco Lopes Sousa, et al. [45] | 710 MSM | Condom and PrEP | ● **Condom:** Proportion of people who used a condom in anal sex to protect from covid-19;  ● **PrEP:** Proportion of people who used PrEP to protect from covid-19; |
| 2021 | Jeremias Salomão Chone et al. [46] | 1,301 MSM | HIV testing , condom and PrEP | ● **Testing:** proportion of participants who got tested for HIV in the last 12 months; ● **Condom:** proportion of participants who condomless sex during social distancing period;  ● **PrEP**: proportion of participants who used PrEP during social distancing period; |
| 2022 | Paula Meireles, et al. [27] | 5167 MSM | HIV testing, condom and PEP | **● Testing:** Proportion of participants who have ever got tested for HIV (stratified according to PrEP eligibility);  **● Condom:** Proportion who have had condomless sex in the past 6 months and sexual partners with unknown HIV status (stratified according to according to follow-up visits).  **● PEP and condom:** proportion who have had condomless sex in the past 6 months and used PEP (stratified according to according to follow-up visits); |
| 2022 | Adriana Curado, et al. [70] | 176 PWID | HIV testing and condom | ● **Testing:** Proportion of participants who have ever got tested for HIV;  ● **Condom:** Proportion of participants who used condom in last sexual intercourse;  ● Prevalence stratified by being HCV+; |
| 2022 | Rhaisa Farias, et al. [68] | 100 Migrants | HIV testing and condom | ● **Testing:** Proportion of participants who have ever got tested for HIV; that got tested less than one year ago; ● **Condom:** Proportion of participants who reported condom use in the last 12 months (always, sometimes or never). |
| 2023 | ECDC [73] | MSM, PWID, SW, migrants and transgender (size not available) | PrEP | **PrEP:** Number of people who got PrEP for the first time in their lives during the last 12 months; Number of people receiving PrEP at least once in reported period; Proportion of PrEP users who are MSM; |
| 2023 | Álvaro Francisco Lopes Sousa, et al. [42] | 1033 Migrants and MSM | HIV testing, condom and PrEP | ● **Testing:** Proportion of participants who ever got tested for HIV in the last 12 months (recent); ● **Condoms:** Proportion of participants who used condoms in last sexual intercourse; ● **PrEP:** Proportion of participants currently on PrEP defined as taking daily PrEP tablet in the last 30 day; ● Prevalence stratified regarding PrEP use and baseline. |
| 2023 | Álvaro Francisco Lopes Sousa, et al. [60] | 712 Migrants and MSM | HIV testing, condom and PrEP | ● **Testing:** Proportion of participants who ever got tested for HIV in the last 12 months; ● **Condoms:** Proportion of participants who reported condom consistency; ● **PrEP:** Proportion of participants currently on PrEP defined as taking daily PrEP tablet in the last 30 day. |
| 2023 | Direção-Geral da Saúde [4] | MSM, PWID, SW and migrants (size not available) | PrEP | ● **PrEP:** Proportion of participants who got PrEP for the first time in their lives during the last 12 months or in their lives; |
| 2023 | Miguel Rocha et al. [44] | MSM, Sex Worker, Migrant, transgender | PrEP | ● **PrEP:** knowledge and use in the last 12 months (stratification based on year of first visit, age range, being born or not in Portugal, being a sex worker or not, sexual orientation, HIV status, per region of the community-based organization where the test was performed, gender); **number of people who got PrEP for the first time or at least one time in the last 12 months stratified by year and region;** |
| 2024 | Direção-Geral da Saúde [3] | MSM, PWID, SW and migrants (size not available) | PrEP | ● **PrEP:** Proportion of participants who got PrEP for the first time in their lives during the last 12 months or in their lives; |
| 2024 | Ricardo Abrantes, et al. [48] | 391 newly HIV positive MSM | HIV testing and condom, PrEP and PEP | ● **Testing:** Proportion of participants HIV screening according to frequency in the last 12 months (more than one time, one time or less than one time and never);  ● **Condom:** Proportion of unprotected anal sex (general, insertive or receptive); anal or vaginal sex with a woman or in trios/group sex in the last 12 months;  ● **PEP:** Proportion of participants who ever used PEP;  ● **PrEP:** Proportion of participants who ever used PrEP; |
| 2024 | Mafalda N. S. Miranda, et al. [47] | 265 Migrants | Condom | ● **Condom:** Proportion of participants who used condom in the last 12 months according to frequency with occasional, regular and sexual workers classified as always, sometimes or never; |
| 2024 | ECDC [65] | SW (not available) | HIV testing, condom and PrEP | ● **Testing:** Proportion of participants who ever got tested for HIV in the last 12 months; ● **Condom:** Proportion using a condom with their most recent client in the last 12 months; ● **PrEP:** number of sex workers receiving PrEP at least once over the reporting period; |
| 2024 | ECDC [43] | 29 365 Migrants and transgender | HIV testing, condom and PrEP | ● **Testing:** Number of migrants who got tested in the last 12 months; ● **Condom:** Proportion of transgender migrants and migrants condom usage last time they had sex among those who had sex in the last 12 months; ● **PrEP:** Proportion of PrEP users who are migrants in the last 12 months |
| 2025 | Rita Dias et al. [57] | 7549 MSM | PrEP, condom, PEP | ● **Condom:** proportion of participants who used condoms in anal sex in the last 12  months (always, almost always, occasionally/rarely/never, did not have anal sex); ● **PEP:** proportion of participants who reported PEP knowledge; who reported PEP use in the last 12 months  **● PrEP:** proportion of participants who reported PrEP knowledge; proportion of participants who reported PrEP use in the last 12 months; |
| 2025 | Ricardo Abrantes et al. [58] | 340 MSM | HIV testing and condom | **● Testing:** proportion of participants according to HIV testing frequency (more than once per year, once per year and never got tested);  **● Condom:** proportion of participants who reported CAI unprotected anal sex (insertive, receptive) or anal or vaginal sex (aggregated) with a woman in the last 12 months; |
