# Supplementary material for: Assessment of knowledge and use of HIV primary and secondary prevention strategies in Portugal: a scoping review
Source: BMC Public Health. 2026 May 7;26:1964. doi: 10.1186/s12889-026-27542-7 (PMC13317048; doi:10.1186/s12889-026-27542-7)
Supplement: Supplementary file 3 — Supplementary Material 3. [file 12889_2026_27542_MOESM3_ESM.docx]

**Assessment of Knowledge and Use of HIV Primary and Secondary Prevention Strategies in Portugal: A Scoping Review**

João Brázia^1,2, §,^ Boxuan Wang^3^, Paula Meireles^4^, Eugenio Valdano^3^, Andreia Sofia Teixeira^1,2^

1. BRAN Lab, Network Science Institute, Northeastern University London, London, UK.
2. LASIGE, Faculdade de Ciências, Universidade de Lisboa, Portugal.
3. Sorbonne Université, INSERM, Institut Pierre Louis d’Epidémiologie et de Santé Publique, F75012, Paris, France.
4. EPIUnit ITR, Instituto de Saúde Pública da Universidade do Porto, Universidade do Porto, Rua das Taipas, n° 135, Porto 4050-600, Portugal.

^§^ Corresponding author: João Brázia, Devon House, 58 St Katharine's Way, London E1W 1LP, [simoesbrazia.j@northeastern.edu](mailto:simoesbrazia.j@northeastern.edu)

**Additional file 2**

This file presents the summary of the initial article selection from PubMed, Web of Science and Scopus (Table 1) and the query search driven in one of the databases (Table 2) according to the requirements established by Preferred Reporting Items for Systematic reviews and Meta-Analyses extension for Scoping Reviews (PRISMA-ScR) Checklist (Table 1 SI-1).

**Table 1** Summary Research Strategy by Source.

|  | **Source** | **Date Searched** | **Number of Results** |
| --- | --- | --- | --- |
| Database Searches Only | PubMed (U.S. National Library of Medicine, National Institutes of Health) | August 15th, 2025 | 269 |
|  | Scopus | August 16th, 2025 | 107 |
|  | Web Of Science | August 18th, 2025 | 279 |
|  | Total | | 655 |
| External Sources | Journal of AIDS and Clinical Research | August 20th, 2025 | 3 |
|  | Intechopen | August 20th, 2025 | 1 |
|  | ECDC | August 20th, 2025 | 8 |
|  | GAT Portugal | August 20th, 2025 | 1 |
|  | Direção Geral de Saúde | August 20th, 2025 | 2 |
| **All Searching Methods Combined** | **Total (Including Duplicates)** | | 670 |
|  | **Total (After Removing)** | | 407 |

**Table 2** Summary of Research strategy on Pubmed.

| **Search** | | **Query** | **Number of Results** |
| --- | --- | --- | --- |
| 1 | "Portugal"[All Fields] OR "portuguese"[All Fields] | | 294,644 results |
| 2 | "prevention"[All Fields] OR "PrEP"[All Fields] OR "PEP"[All Fields] OR "condom"[All Fields] OR "Pre-exposure Prophylaxis"[All Fields] OR "post exposure prophylaxis"[All Fields] OR "Testing"[All Fields] OR "test"[All Fields] OR "screening"[All Fields] OR ("unprotected"[All Fields] AND ("sex"[All Fields] OR "intercourse"[All Fields])) OR "condomless"[All Fields] | | 5,516,015 results |
| 3 | "msm"[All Fields] OR "gay"[All Fields] OR "Men who have sex with men"[All Fields] OR "immigrants"[All Fields] OR "migrants"[All Fields] OR "sex workers"[All Fields] OR "SW"[All Fields] OR "prostitutes"[All Fields] OR "transgender"[All Fields] OR "persons who inject drugs"[All Fields] OR "PWID"[All Fields] OR "key population"[All Fields] | | 180,894 results |
| 4 | "HIV"[All Fields] OR "Human immunodeficiency virus"[All Fields] | | 451,468 results |
| 5 | #1 AND #2 AND #3 AND #4 | | 287 |
| 6 | #5 AND (from 2008 to 2025) AND (English[Language] OR Portuguese[Language]) | | 269 |

**Table 3** Summary of Research strategy on Scopus.

| **Search** | **Query** | **Number of Results** |
| --- | --- | --- |
| 1 | TITLE-ABS-KEY ( "Portugal"  OR "portuguese" ) | 143,169 results |
| 2 | TITLE-ABS-KEY ( "prevention" OR "PrEP" OR "PEP" OR "condom" OR "Pre-exposure Prophylaxis" OR "post-exposure Prophylaxis" OR "testing" OR "test" OR "screening" OR ( "unprotected" AND ( "sex" OR "intercourse" ) ) OR "condomless" ) | 13,237,551 results |
| 3 | TITLE-ABS-KEY ( "MSM" OR "gay" OR "men who have sex with men" OR "immigrants" OR "migrants" OR "sex workers" OR "SW" OR "prostitutes" OR "transgender" OR "persons who inject drugs" OR "PWID" OR "key population" ) | 438,754 results |
| 4 | TITLE-ABS-KEY ( "HIV" OR "Human immunodeficiency virus" ) | 586,970 results |
| 5 | #1 AND #2 AND #3 AND #4 | 122 |
| 6 | #5 AND (PUBYEAR > 2007 AND PUBYEAR < 2026) AND AND ( LIMIT-TO ( LANGUAGE , "English" ) OR LIMIT-TO ( LANGUAGE , "Portuguese" ) ) | 107 |

**Table 4** Summary of Research strategy on Web of Science.

| **Search** | **Query** | **Number of Results** |
| --- | --- | --- |
| 1 | ALL=("portugal" OR "portuguese" ) | 1,008,413  results |
| 2 | ALL=("prevention" OR "PrEP" OR "PEP" OR "condom" OR "Pre-exposure Prophylaxis" OR "post-exposure Prophylaxis" OR "Testing" OR "test" OR "screening" OR ("unprotected" AND ("sex" OR "intercourse")) OR "condomless") | 7,390,777 results |
| 3 | ALL=("msm" OR "gay" OR "Men who have sex with men" OR "immigrants" OR "migrants" OR "sex workers" OR "SW" OR "prostitutes" OR "transgender" OR "persons who inject drugs" OR "PWID" OR "key population") | 1,045,215  results |
| 4 | ALL=("hiv" OR "human immunodeficiency virus") | 532,089  results |
| 5 | #1 AND #2 AND #3 AND #4 | 317 |
| 6 | #1 AND #2 AND #3 AND #4 AND English or Portuguese (Languages) AND  2008-01-01 to 2025-08-18 (Publication Date) | 279 |
